# Estimating the efficacy of pharmacogenomics over a lifetime

**DOI:** 10.3389/fmed.2023.1006743

**Published:** 2023-10-31

**Authors:** Zhan Ye, John Mayer, Emili J. Leary, Terrie Kitchner, Richard A. Dart, Murray H. Brilliant, Scott J. Hebbring

**Affiliations:** Marshfield Clinic Research Institute, Marshfield, WI, United States

**Keywords:** Pharmacogenenomics and personalised medicine, electronic health record (EHR), drug responce, precision medicine, individualized medicine

## Abstract

It is well known that common variants in specific genes influence drug metabolism and response, but it is currently unknown what fraction of patients are given prescriptions over a lifetime that could be contraindicated by their pharmacogenomic profiles. To determine the clinical utility of pharmacogenomics over a lifetime in a general patient population, we sequenced the genomes of 300 deceased Marshfield Clinic patients linked to lifelong medical records. Genetic variants in 33 pharmacogenes were evaluated for their lifetime impact on drug prescribing using extensive electronic health records. Results show that 93% of the 300 deceased patients carried clinically relevant variants. Nearly 80% were prescribed approximately three medications on average that may have been impacted by these variants. Longitudinal data suggested that the optimal age for pharmacogenomic testing was prior to age 50, but the optimal age is greatly influenced by the stability of the population in the healthcare system. This study emphasizes the broad clinical impact of pharmacogenomic testing over a lifetime and demonstrates the potential application of genomic medicine in a general patient population for the advancement of precision medicine.

## Introduction

US Food and Drug Administration (FDA) currently recognizes over 60 genes with variants that can predict response/efficacy for over 190 drugs (1, 2). Whereas some of these genes may have somatic variants that predominantly influence antineoplastic medications, nearly half are recognized for their germline variants, including common variants. Population-based studies have repeatedly shown that over 90% of a general patient population may carry one or more of these common variants (3–5). For example, the minor allele frequency (MAF) for reduced function alleles in *CYP2C19*, variants that inhibit the bioactivation of antiplatelet drug clopidogrel, range between 15%–30% in Asian and Caucasian populations (CYP2C19*2 allele), can be nonexistent in those with African ancestries (CYP2C19*3 allele), and 15% in East Asians (CYP2C19*3 allele) (6). Reduced function carriers treated with clopidogrel do not have sufficient antiplatelet activity and are thus at increased risk for on-treatment stent thrombosis and cardiovascular causes of death (7, 8). Beyond clopidogrel, *CYP2C19* genotype may also contribute to the response of at least 15 additional medications (1, 2).

Arguably the gene with the largest single impact on variation in drug response is *CYP2D6* due to multiple common functional variants that influence the metabolism and/or bioactivation of about 25% of medications including 40 different drugs recognized by the FDA and CPIC (1, 2). The locus containing *CYP2D6* is genetically complex with both gain and loss-of-function alleles (9). In Caucasian populations, the loss-of-function alleles are the predominant form. Codeine is one drug metabolized by *CYP2D6* that has received significant attention in the literature as seen by case-reports (10–12). Codeine is a prodrug that requires CYP2D6 for bioactivation (codeine ➔ morphine). Individuals who are poor metabolizers for CYP2D6 often receive little benefit from codeine whereas those who are ultra-rapid metabolizers are at increased risk for morphine toxicity. Instead of adopting pharmacogenomic testing to mitigate potential risks (13, 14), codeine is now being used less in pediatric populations due to FDA Black Box warnings (15, 16).

Even though governmental agencies and the research community have recognized the importance of *CYP2D6*, *CYP2C19*, and other relevant genes for drug prescribing, the adoption of pharmacogenomic testing in clinical care has been slow. This owes in part to unawareness and skepticism from the larger medical community (17) and concerns regarding high costs for alternative medications when the primary drug may be contraindicated. If pharmacogenomic testing is applied, it is often during point-of-care and limited to the gene(s) relevant to the drug being prescribed. An alternative approach would include pre-emptive genetic testing, which healthcare payers in the United States and most of the countries around the world often do not support. Pre-emptive pharmacogenomic testing could provide lifelong benefits, but data attesting to its value are sparse since prospective studies would require years, if not decades, of follow-up. To address this limitation, we conducted a retrospective study in an unselected population with life-long medical record data to understand the potential clinical impact of pharmacogenomics on drug prescribing over a lifetime. Such findings may be of great importance when implementing pharmacogenomic testing and precision medicine for a general patient population.

## Methods

To quantify the potential clinical impact of pharmacogenomics over a lifetime, we sequenced the genomes of deceased patients from Marshfield Clinic’s Personalized Medicine Research Project (PMRP). PMRP is a population cohort of over 20,000 individuals and has been described previously (18–20). Over 1,800 PMRP participants were deceased at the beginning of this study, including approximately 1,000 with extensive lifetime medical histories that cover over 30 years of longitudinal and nearly comprehensive electronic health record (EHR) data. Of these 1,000 deceased individuals, 300 were randomly selected resulting in 161 females and 139 males. All participants consented under an approved IRB protocol for research prior to death. Cause of death was determined by manual chart review of medical records by a trained study coordinator and physician (Supplementary Table S1). Prescribed medications were extracted from structured patient EHR data maintained in a relational database at Marshfield Clinic.

All patients were whole genome sequenced by Complete Genomics (Mountain View, CA). Five to ten nanograms of DNA was fragmented (400–600 bp) and mate-paired reads of 35 nucleotides of either end of the fragments were determine using a nanoarray-based short-read sequencing-by-ligation technology (21). The average mate gap length was 300 bp. Variant calling was performed using Complete Genomic’s proprietary software (version 2.2) and NCBI reference genome build 37. Quality scores for each variant were calculated as −10 × log10[*P*(call is true)/*P*(call is false)] measuring the confidence in the variant call. Homozygous variants with quality scores less than 20 and heterozygous variants with quality scores less than 40 were excluded from downstream analysis as low quality variants. A minimum read-depth of 10 was required for variant calling. Emphasis was placed on germline variants mapped to 33 pharmacogenes recognized by the FDA to affect drug response for 118 medications (Supplementary Table S2) (1, 2). A total of 17 pharmacogenomic SNPs were polymorphic, whereas the remaining loci were monomorphic, had insufficient read-depth, or not analyzed due to technical challenges when calling variants (e.g., structural *CYP2D6* variants).

## Results

In the 300 deceased patients, we identified 17 clinically significant variants in nine pharmacogenes (Table 1). For example, 100 patients (33%) carried one or more known loss-of-function *CYP2D6* SNPs (i.e., *4, *6, and/or *10 alleles). It should be noted that the *CYP2D6* locus is a genetically complex region containing pseudogenes, deletions, duplications, and/or rearrangements (9, 14) that were not assayed. When considering all pharmacogenes, 278 deceased Marshfield Clinic patients (93% of total population) carried at least one clinically significant pharmacogenomic variant consistent with previous observations (3–5).

**Table 1:** List of pharmacogenomic variants identified from whole-genome sequencing of 300 deceased patients.

Gene	Chr	Position	Nucleotide	Variant ID	Allele	Homozygotes/heterozygotes
*CFTR*	7	117227860	G > A	rs75527207	Gly551Asp	0/1
*CYP2C19*	10	96534922	G > C	rs17878459	*2B	0/9
*CYP2C19*	10	96541616	G > A	rs4244285	*2	10/75
*CYP2C9*	10	96702047	C > T	rs1799853	*2, *24, *35	5/56
*CYP2C9*	10	96741053	A > C	rs1057910	*3 and *18	3/46
*CYP2D6*	22	42524947	C > T	rs3892097	*4	9/45
*CYP2D6*	22	42525086	A > -	rs5030655	*6	0/4
*CYP2D6*	22	42526694	G > A	rs1065852	*10	12/73
*DPYD*	1	97547947	T > A	rs67376798	Asp949Val	0/5
*DPYD*	1	97915614	C > T	rs3918290	Splice donor	0/5
*F5*	1	169519049	C > T	rs6025	Arg534Gln	0/14
*SLCO1B1*	12	21331549	T > C	rs4149056	*5	7/82
*TPMT*	6	18130918	T > C	rs1142345	*3A	2/27
*TPMT*	6	18139228	C > T	rs1800460	*3A	2/26
*TPMT*	6	18143955	C > G	rs1800462	*2	0/2
*VKORC1*	16	31104878	G > A	rs9934438	*2	40/136
*VKORC1*	16	31107689	C > T	rs9923231	*2	40/138

**Figure 1:**
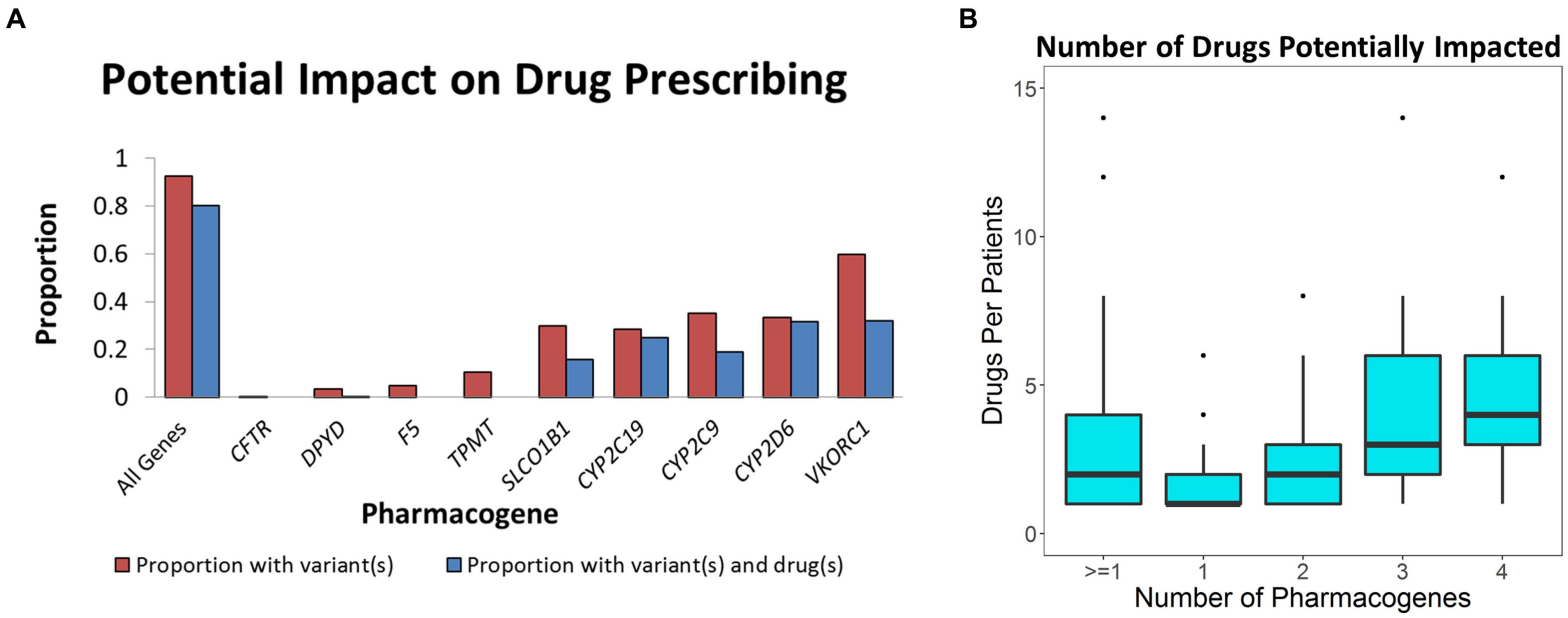
Impact of pharmacogenomic testing on drug prescribing. **(A)** Defined in red is the proportion of population with known pharmacogenomic variants in the corresponding pharmacogenes. Blue represents the percent of the population that was given a drug that may have been impacted by the relevant variants. **(B)** Box plot describing number of relevant drugs given to those who had clinically significant variants in one to four pharmacogenes.

According to FDA drug labeling, variants in *CYP2D6* can impact the metabolism of at least 40 different medications (1). Of these, there is evidence that 26 different medications were prescribed impacting nearly all 100 patients with *CYP2D6* variants. When considering all drugs and genes, 80% (241 patients) were prescribed any one of 42 different medications (Figure 1A) representing 634 unique drug-patient incidences which may have been impacted by these variants. For the 241 patients with a clinically significant pharmacogenomic variant, the mean and median number of drugs given to these patients that may have been affected was 2.8 and 2.0, respectively (Figure 1B). In one case, a patient with variants in three genes was given 14 different medications that may have been affected by their genetic profile.

Individuals with variants in these genes may be at risk for an adverse drug event. For example, heterozygotes for SLCO1B1*5 have a moderate risk whereas homozygotes (*5/*5) are at high risk for myopathies if treated with simvastatin (2, 22–24). Manual chart review of clinical notes for all 154 patients having taken simvastatin identified 21 with symptoms consistent of simvastatin induced myopathies. Of the four *5/*5 homozygotes given simvastatin, three had documented drug complications to simvastatin including two with myopathies that may have been prevented had they been tested.

**Figure 2:**
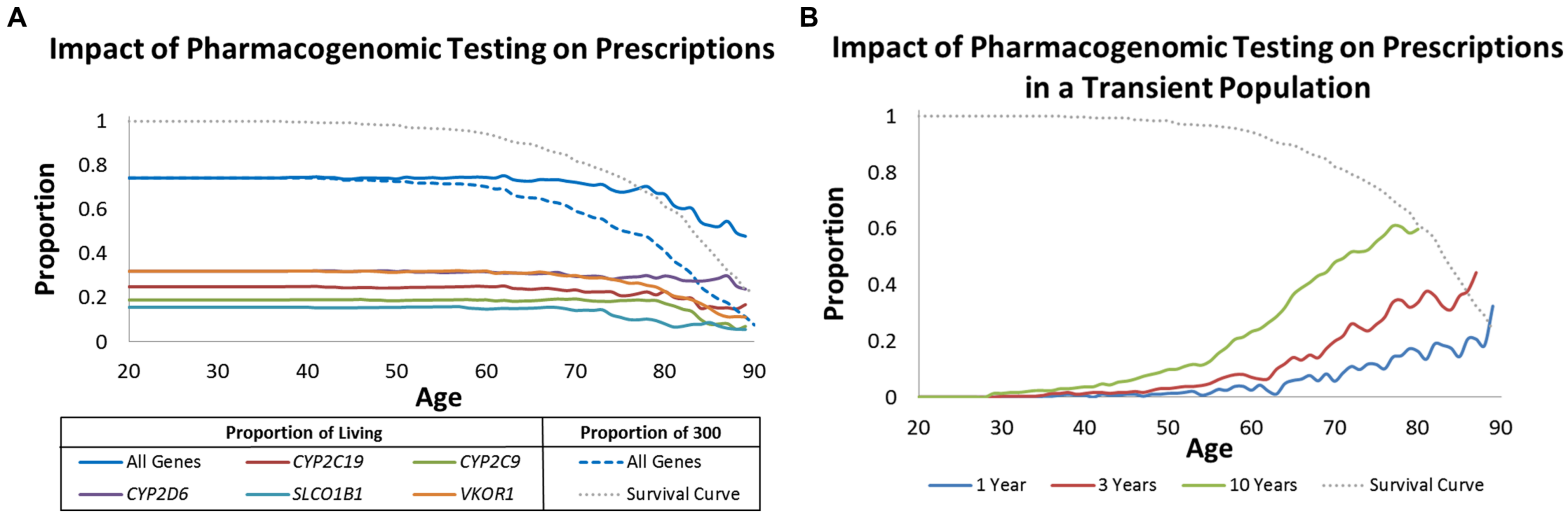
Impact of pharmacogenomic testing over time. **(A)** Proportion of living patients (solid lines) and proportion of all 300 patients (dashed line) that may benefit from pharmacogenomic testing when considering age at prescription. Results are provided by gene and all genes combined. **(B)** Proportion of living patients that may benefit from pharmacogenomic testing when considering age of prescription but modeled to reflect a transient population lost to follow-up in 1, 3, and 10 years. Graphed in **(A,B)** is a survival curve for all 300 patients (grey dotted line).

Focusing on deceased patients allowed us to quantify at what age preemptive pharmacogenomic testing may provide the broadest impact. We evaluated this question from three real-world perspectives. One perspective is to consider what proportion of the 300 patients could benefit from pharmacogenomic testing prior to drug(s) being prescribed. In this case, the numerator represented number of patients who could have benefited from pharmacogenomic testing whereas the denominator was always 300. Under this model, approximately 80% of the population could have benefited from pharmacogenomic testing up until age 50. It was not until after age 50 was there diminished value similar to the survival curve of the population (Figure 2A).

The second perspective is to evaluate the optimal age of pharmacogenomic testing in living populations. For example, is it practical to test an 80 year old if he/she may have already been exposed to many medications and remaining life expectancy is predicted to be short? Under this model, the numerator is the same as before, but the denominator changes according to who is living at a given age. In comparison to the first model, the value of pharmacogenomic testing in living populations far exceeded age 50. It was not until well after age 70 that the benefit for pharmacogenomic testing of living individuals continuously decreased with age (Figure 2A).

The last real-world scenario was to determine potential benefit for pharmacogenomic testing in living but transient populations where patients may be lost to follow-up within 1, 3, and 10 years. Even though nearly 80% of the living population would have benefitted from pharmacogenomic testing by age 50 (Figure 2A), only 1%, 3%, and 10% would have benefitted if all patients were lost to follow-up in 1, 3, and 10 years, respectively (Figure 2B). The greatest benefit in a transient population was observed after age 70; an age group that is likely geographically stable and likely to be exposed to multiple drugs.

## Conclusion

This study demonstrates the potential impact of pharmacogenomics over a lifetime. The vast majority of individuals carried clinically actionable variants across multiple pharmacogenes. Nearly 80% of a general patient population could have benefited from pharmacogenomic testing over a lifetime. The ideal time to conduct pharmacogenomic testing is prior to the administration of relevant medications so that the right drug at the optimal dose can be given. Based on our population, pharmacogenomic testing for optimal effectiveness should be conducted prior to age 50 but approximately 70% of living 70 year olds can have benefit if tested. The impact of this result though was greatly influenced by the stability of the patient population.

Results from this study is likely an underrepresentation of the actual lifelong impact of pharmacogenomics in patient populations. Aside from technical limitations when typing some variants (e.g., copy number variants in *CYPD2D6*), there were numerous drug-gene pairs that were not evaluated. We limited the scope of our analysis to only those drug-gene pairs recognized by FDA and CPIC to ensure high fidelity of the clinical interpretations of our results, but we recognize there are additional drug-gene pairs evaluated in clinical practice and tested by reputable companies (25). Even though our analysis was limited to databases maintained by FDA and CPIC, these databases are ever changing. For example, this study included the evaluation of patients with variants in *SLCO1B1* and simvastatin, but CPIC expanded guidelines as recently as 2022 to three different genes and seven statin medications (26). CPIC also recently provided pharmacogenomic guidelines pertaining to 15 NSAIDs, including recommendations for one of the most commonly used NSAIDs, ibuprofen (27). A comprehensive evaluation of patients taking ibuprofen would have been further complicated since this medications is available “over the counter.” Given the ever growing body of knowledge in pharmacogenomics, it is not unreasonable to predict much greater than 80% of a patient population could benefit from pharmacogenomic testing because they will be given many more than three medications that will be influenced by their genetic profiles. This likelihood would also result in patients benefiting from pharmacogenomic testing at younger ages.

Although our results suggest most individuals could benefit from pharmacogenomic testing, patients sequenced were primarily older individuals of European descent from a rural community. Different ethnicities have different pharmacogenomic profiles, and the impact of rare variants for some of these genes is currently unknown. Furthermore, drug prescribing patterns will be influenced by individual treating physicians, different healthcare institutions, and patient social economic status.

It is well accepted that pharmacogenomic variants impact drug efficacy, risk for adverse drug events, or both. Although our limited manual chart review of patients on simvastatin uncovered significant benefits of pharmacogenomic testing, it would have been impractical to manually evaluate adverse drug events and therapeutic outcomes for all patients. There are also no existing tools to programmatically extract this information in a comprehensive and high throughput manner from an EHR. Given most pharmacogenomic variants have moderate affects, we would have been underpowered to formally evaluate many drug-gene pairs. It is anticipated that some of these challenges will likely be addressed by larger patient cohorts that link extensive genomic data with clinical outcomes and medication use *via* EHR data in multi-ethnic cohorts with over 1 million participants at all ages [e.g., *All-of-Us* Research Program (28)]. Expanding this line of research to larger diverse populations will allow for a more comprehensive evaluation of both clinical outcomes and economic impact of pharmacogenomic testing over a lifetime.

In conclusion, this study firmly demonstrates nearly everyone carries a clinically actionable pharmacogenomic variant that may impact one or more medications taken over a lifetime. By expanding pharmacogenomic testing across all age groups, everyone can benefit from the promise of precision medicine.

## Data availability statement

Data presented in this study has been deposited in dbGAP (https://www.ncbi.nlm.nih.gov/gap/; phs003454).

## Ethics statement

The studies involving human participants were reviewed and approved by Marshfield Clinic Research Institute Internal Review Board. The patients/participants provided their written informed consent to participate in this study.

## Author contributions

MB procured funding for study activities. TK helped manage study activities. JM and ZY conducted data analysis. EL and RD conducted chart review. SH was responsible for all aspects of the study including writing the first draft. All authors contributed to the article and approved the submitted version.
