# Supplementary material for: Estimating the efficacy of pharmacogenomics over a lifetime
Source: Front Med (Lausanne). 2023 Oct 31;10:1006743. doi: 10.3389/fmed.2023.1006743 (PMC10645151; doi:10.3389/fmed.2023.1006743)
Supplement: Supplementary file 1 [file Table_1.DOCX]

| **Supplemental Table 1:** Demographics of the 300 deceased Marshfield Clinic patients | | |
| --- | --- | --- |
| Cause(s) of Death* | Males (%)  n=139 | Females (%)  n=161 |
| Accident (fall, burn, motor vehicle) | 4.0 | 1.9 |
| Cardiovascular (CHF, cardiomyopathy, aneurysm) | 33 | 38 |
| Cancer (pancreatic, breast, lymphoma) | 32 | 30 |
| Renal (acute and chronic kidney disease) | 8.0 | 9.9 |
| Infection (pneumonia and sepsis) | 10 | 9.3 |
| Neurologic (stroke, Alzheimer's, Parkinson’s) | 16 | 17 |
| Pulmonary (COPD, fibrosis, interstitial lung disease) | 19 | 21 |
| Other (liver disease, myelodysplastic syndrome, colitis) | 8.0 | 3.1 |
| Unknown | 12 | 13 |
|  |  |  |
| Self-Reported Ancestry |  |  |
| White European | 99.3 | 99.4 |
| Native American | 0.7 | 0.6 |
|  |  |  |
|  | [Mean/Median] | [Mean/Median] |
| Age of death (years) | 81/84 | 81/84 |
| Years of EHR data | 31/31 | 31/31 |
|  |  |  |
| (*) Percentages may add up to greater than 100% as some patients may have died due to multiple conditions; examples of cause of death are provided in parentheses. CHF, chronic heart failure; COPD, chronic obstructive pulmonary disease. | | |
